# Health Tract, No. 291

**Published:** 1867-05

**Authors:** 


HEALTH TRACT, No. 291.

### WRONG END FOREMOST.

"Some persons get up wrong end foremost," was the suggestive remark the other day of a maiden lady, whose sterling worth, the most obtuse would discover after a very short acquaintance. Right merrily do the summer birds twitter in the spring, and the little lambkins too, skip around of a May morning, as if well pleased with themselves and all the world; it is left only to a certain class of human brutes, to rise from their beds in a humor, ugly, mischievous and sometimes even devilish, instead of opening their eyes to the beautiful morning with hearts overflowing with humble thankfulness that they have arisen from the counterpart of the grave,with their property, their reason, their health, and their lives all preserved to them, by the watchful care of HIM whose eye never slumbers or sleeps—they begin the day with angriness, with impatient fault-findings; ready to fly in a passion at every trivial occurrence not precisely in accordance with their wishes; not content to indulge in their unamiablenesses in their chambers, they bring them down to the breakfast table—the husband to vent them on the uncomplaining and patient wife, the young man in surly orders to the servants, and the budding girl to cast a fretful glance on the good things before her, forgetting that they would have been sweet indeed, were it not for her late hours, her neglect of daily exercises in the open air and her indulgences in eating unregularly, in eating "between times," and in taking hearty and late meals. The pig, when he comes to his trough, quirls his little tail in glad anticipations of good things from the slop tub; and the very sight of a bone causes the faithful dog to wag the extension of his corporosity in seeming gladness; it is for men, at least the class which belongs to the order of uncultured brutes, to whine and growl and fidget about until the meal is nearly finished, then their brutality is relaxed; they gradually rise to the self complacent and the amiable, and if they had tails, no doubt would wag them with delight like any other dog. This "getting up wrong end foremost," arises from one or two causes, from an inherent snaky, spiteful nature, or from an acquired quality arising from bad habits of life, late hours, gormandizing after sun-down, as if the chief end of man were to stuff his worthless paunch with thrice what it was intended to hold, and in the indulgence of hateful fault-findings, of belittling querulousness, and of those fretful whinings which always indicate a weak mind and a grovelling nature.

